# Genetic Dissection of Hypertrophic Cardiomyopathy with Myocardial RNA-Seq

**DOI:** 10.3390/ijms21093040

**Published:** 2020-04-25

**Authors:** Jun Gao, John Collyer, Maochun Wang, Fengping Sun, Fuyi Xu

**Affiliations:** 1 Department of Genetics, Genomics, and Informatics, University of Tennessee Health Science Center, Memphis, TN 38163, USA; jgao21@uthsc.edu; 2 Institute of Animal Husbandry and Veterinary Science, Shanghai Academy of Agricultural Sciences, Shanghai 201106, China; 3 Department of Pediatrics, University of Tennessee Health Science Center, Memphis, TN 38163, USA; jcollyer@uthsc.edu; 4 College of Chemistry, Chemical Engineering, and Biotechnology, Donghua University, Shanghai 201620, China; wangmaochun0815@163.com

**Keywords:** hypertrophic cardiomyopathy, RNA-seq, pathogenic variants, differential gene expression, gene network

## Abstract

Hypertrophic cardiomyopathy (HCM) is an inherited disorder of the myocardium, and pathogenic mutations in the sarcomere genes myosin heavy chain 7 (*MYH7*) and myosin-binding protein C (*MYBPC3*) explain 60%–70% of observed clinical cases. The heterogeneity of phenotypes observed in HCM patients, however, suggests that novel causative genes or genetic modifiers likely exist. Here, we systemically evaluated RNA-seq data from 28 HCM patients and 9 healthy controls with pathogenic variant identification, differential expression analysis, and gene co-expression and protein–protein interaction network analyses. We identified 43 potential pathogenic variants in 19 genes in 24 HCM patients. Genes with more than one variant included the following: *MYBPC3*, *TTN*, *MYH7*, *PSEN2*, and *LDB3*. A total of 2538 protein-coding genes, six microRNAs (miRNAs), and 1617 long noncoding RNAs (lncRNAs) were identified differentially expressed between the groups, including several well-characterized cardiomyopathy-related genes (*ANKRD1*, *FHL2*, *TGFB3*, *miR-30d*, and *miR-154*). Gene enrichment analysis revealed that those genes are significantly involved in heart development and physiology. Furthermore, we highlighted four subnetworks: mtDNA-subnetwork, DSP-subnetwork, MYH7-subnetwork, and MYBPC3-subnetwork, which could play significant roles in the progression of HCM. Our findings further illustrate that HCM is a complex disease, which results from mutations in multiple protein-coding genes, modulation by non-coding RNAs and perturbations in gene networks.

## 1. Introduction

Hypertrophic cardiomyopathy (HCM) is a global, inherited cardiovascular disease that can lead to arrhythmia, heart failure and even sudden cardiac death [1,2]. It is characterized by ventricular hypertrophy (usually the left ventricle), small ventricular cavity, and decreased ventricular diastolic compliance. The prevalence of HCM is estimated at 0.2% of the general population by early echocardiographic studies [2]. The utilization of cardiac magnetic resonance imaging (MRI) and genetic testing has increased recognition and precise clinical diagnosis of HCM [3,4]. Genetic testing, though, has revealed that some mutation carriers do not exhibit obvious clinical symptoms of HCM. It is still possible for a carrier to later demonstrate HCM symptoms and to pass these potentially pathogenic mutations on to offspring [5]. With the use of these more sophisticated technologies, the prevalence of HCM should be modified to 0.5% or even greater [2]. 

Variants in sarcomere and sarcomere-associated protein genes represent the greatest genetic contributor to HCM cases. Previous studies have shown that pathogenic mutations in myosin heavy chain 7 (*MYH7*) and cardiac myosin-binding protein C (*MYBPC3*) account for 60%–70% of identified HCM cases [6]. About 1%–5% of HCM and HCM-like phenotypes can be attributed to pathogenic mutations in other sarcomere protein genes (e.g., *TNNT2*, *TNNI3*, *TPM1*, *MYL2*, *MYL3*, *ACTC1*, and *TNNC1*) and some non-sarcomere protein genes (e.g., Z-disk and calcium-handling proteins) [6,7].

HCM represents a genetically complex and heterogeneous disease [7]. High throughput sequencing technologies have greatly reduced the cost of genetic testing in familial HCM screening, and numerous disease-causing variants had been identified and archived [8,9,10]. Related individuals carrying an identical mutation in the same gene, may display different HCM phenotypes and experience different clinical outcomes [11], suggesting that novel causative or modifier genes exist or that non-coding RNAs, such as microRNA (miRNA) and long noncoding RNA (lncRNA). Furthermore, the complex HCM phenotype may result, not from monogenic or even digenic perturbation, but from the synergy of a complex gene co-expression network. With our current understanding of cardiomyopathy genetics, genotype–phenotype correlation may not be obvious in HCM patients. Since the genetic modifier and pathway/co-expression perspectives are both possibilities, distinguishing pathogenic genes and variants and establishing clear genotype–phenotype correlations remain challenging. 

Previously, Gu *et al*. [12] generated 37 myocardial RNA-seq data from HCM patients and healthy donors. Their research mainly focused on RNA-seq data quality evaluation, novel lncRNA gene prediction, and differential expression analysis. The results demonstrated that a high-quality dataset was obtained and hundreds of differentially expressed genes (DEGs) were identified between HCM patients and healthy controls. In this study, we systemically analyzed this dataset with a different perspective. This included the identification of functional variants, differentially expressed coding and noncoding genes, and interpretation of their potential functional roles associated with HCM. In addition, we also performed weighted gene co-expression network analysis (WGCNA) and protein–protein interaction (PPI) network analysis to identify a gene network that was associated with HCM clinical traits.

## 2. Results

### 2.1. Statistics of RNA-Seq Data

The myocardial RNA-seq included 28 HCM patients and 9 healthy controls. For HCM samples, *MYBPC3* mutations were previously identified in 10 samples and *MYH7* mutations in eight (Table 1). The other 10 remained genetically undiagnosed (Table 1). The RNA-seq data generated 4.3 billion reads with an average of 116.5 million per sample. After quality control, read mapping, variant calling, and filtering, ~0.75 million variants, including single nucleotide polymorphisms (SNPs) and insertions and deletions (Indels), on average, were identified for each sample. Approximately 34,000 variants were located in exonic regions. The summary statistics of the RNA-seq data are listed in Table 2.

### 2.2. Pathogenic Variants and Genes Prioritization

**Figure 1:**
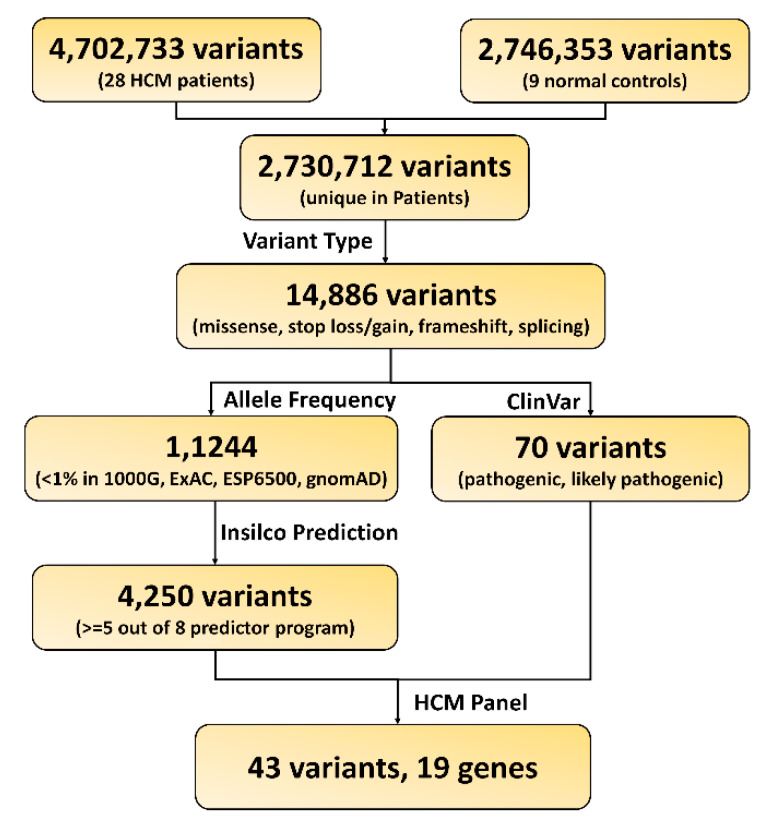
Workflow for functional variants and gene prioritization.

**Figure 2:**
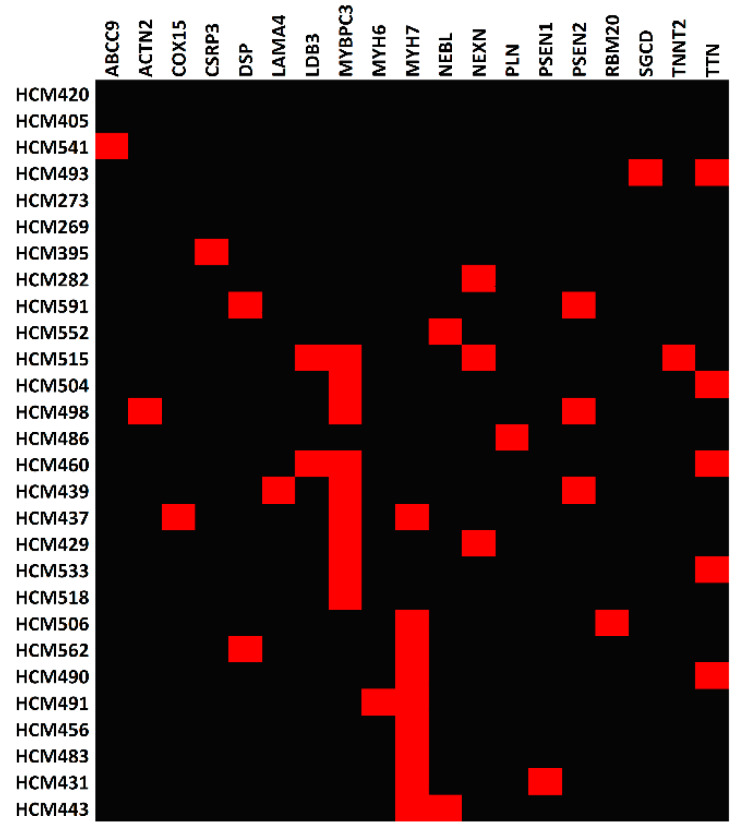
Heatmap of the pathogenic genes across hypertrophic cardiomyopathy (HCM) patients. Red rectangles represent HCM patients (rows) who carry pathogenic variant(s) in the corresponding gene(s) (columns).

In order to identify the pathogenic variants and genes responsible for the development of HCM, we applied a series of bioinformatics filters to the exonic variants identified in the HCM patients (Figure 1). We identified over 4000 candidate variants that matched our criteria: 1) variants only existed in the HCM patients, 2) variants classified as nonsynonymous, stop-gain, stop-loss, frameshift, or splice altering, 3) variants with minor allele frequency less than 0.01 in human populations, and 4) variants predicted to be deleterious in silicon. In order to further narrow down the candidate lists, we filtered the above variants with the 73-gene HCM panel (Supplementary Data 1). This resulted in a total of 43 potential candidate variants, and 19 genes were prioritized (Figure 2 and Table 3). Therefore, our following analysis and discussions were mainly focused on those 19 genes, and the 4000 candidate variants were also listed in Supplementary Data 2 for review. 

For the 10 patients with previously identified *MYBPC3* functional mutations, we found nine *MYBPC3* variants (five frameshift variants, three stop-gain variants, and one splicing variant) in nine patients (Figure 2 and Table 3). HCM518 had a *MYBPC3* mutation in isolation, and *MYBPC3*, *NEXN*, *LDB3*, and *TNNT2* variants were identified in HCM515. For the remaining *MYBPC3* HCM samples, variants were also found in the following other cardiomyopathy genes: *ACTN2*, *LAMA4*, *LDB3*, *NEXN*, *PSEN2*, *TNNT2*, and *TTN*. The remaining patient, HCM486, did not have an identifiable *MYBPC3* variant, but an unreported mutation (c.T106C:p.C36R) was found in *PLN*. 

Seven missense *MYH7* variants were found in nine samples (Figure 2 and Table 3), with two (HCM456 and HCM483) having a *MYH7* mutation in isolation and one (HCM437) possessing *MYH7*, *MYBPC3*, and *COX15* variants. For the remaining *MYH7* HCM samples, variants in the following other cardiomyopathy genes were also found: *DSP*, *MHY6*, *NEBL*, *PSEN1*, *RBM20*, and *TTN*. 

We identified eight candidate variants in six genetically undiagnosed patients (Figure 2 and Table 3). These variants include *ABCC9* (c.T2935C:p.W979R) in HCM541, *SGCD* (c.A845G:p.Q282R) and *TTN* (c.C28730T:p.P9577L) in HCM493, *CSRP3* (c.A585C:p.X195C) in HCM395, *NEXN* (c.C643T:p.R215C) in HCM282, *DSP* (c.G6119A:p.R2040Q) and *PSEN2* (c.G640T:p.V214L) in HCM591, and *NEBL* (c.G561C:p.Q187H) in HCM552. However, we were unable to identify any variants in the remaining four genetically undiagnosed patients (Figure 2).

### 2.3. Identification of DEGs between HCM Patients and Healthy Controls

**Figure 3:**
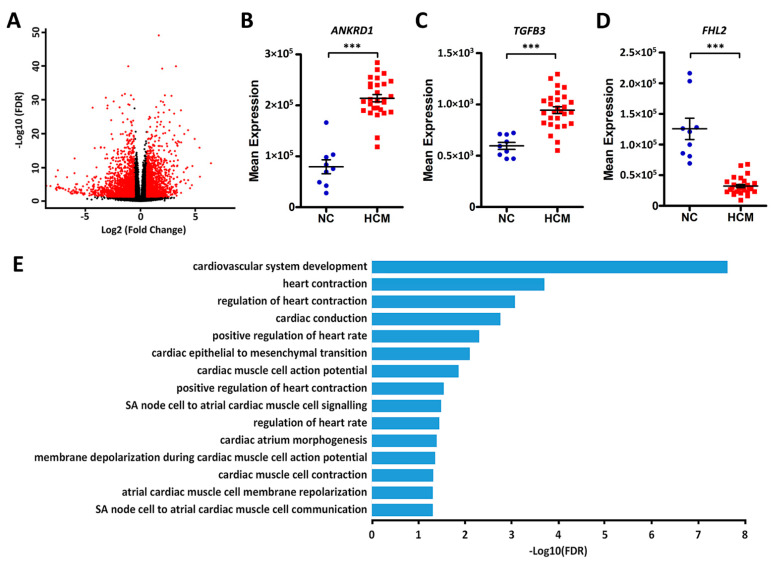
Differentially expressed protein-coding genes between HCM patients and normal controls. (**A**) Volcano plot of the differentially expressed genes (DEGs). The *x*-axes show the log2 transformed fold change, and the *y*-axes show the log10 transformed false discovery rate (FDR). Red dots are DEGs with an FDR < 0.05 and fold change > 1.5. (**B–D**) Dot plots of the expression levels of *ANKRD1*, *TGFB3*, and *FHL2* in normal controls (NC) and HCM patients, ***FDR < 0.001. (**E**) Bar plots of the significantly enriched cardiovascular-related Gene Ontology (GO) terms for the differentially expressed protein-coding genes.

In order to assess the DEGs, we compared gene expression differences between HCM and normal controls by using DEseq2 software. Based on the cut-off threshold (false discovery rate (FDR) < 0.05 and fold change > 1.5), a total of 4161 genes were identified as DEGs (Figure 3A and Supplementary Data 3), of which 2538 are protein-coding genes, 6 are miRNAs, and 1617 are lncRNAs.

**Figure 4:**
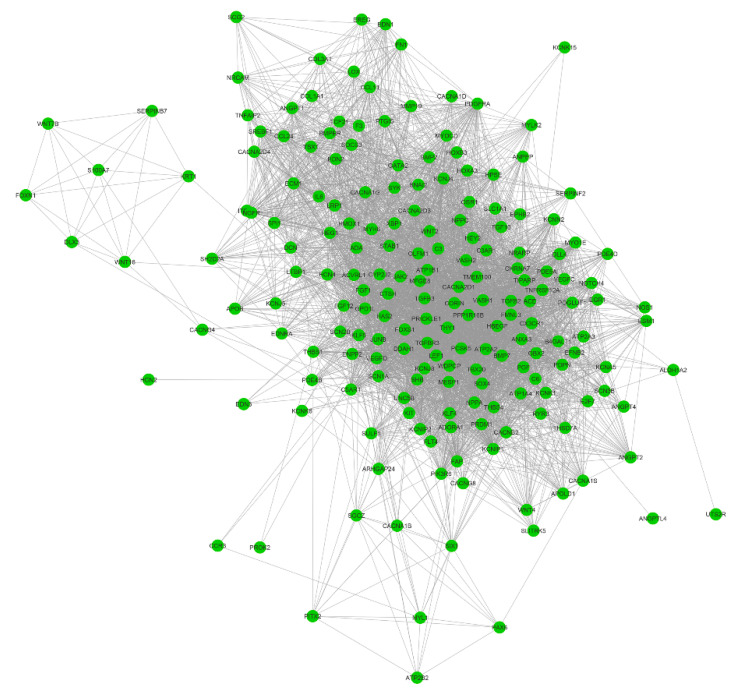
Correlation networks for the cardiovascular-related differentially expressed protein-coding genes. The Pearson correlation coefficient analysis was calculated across the 192 cardiovascular-related genes. Nodes represent genes, and edges represent correlations between two genes (FDR < 0.01).

We did not find any significant expression differences for *MYBPC3* and *MYH7* between the groups. However, several genes that have been implicated in cardiomyopathy did demonstrate large differences between normal controls and HCM patients, including *ANKRD1*, *FHL2*, and *TGFB3* (Figure 3B–D). In order to interpret the differentially expressed protein-coding genes, we performed gene over-representation analysis for Gene Ontology (GO) and Kyoto Encyclopedia of Genes and Genomes (KEGG) pathways. The results demonstrated that these genes are largely involved in regulating cardiovascular system development and cardiac physiology. (Figure 3E, Supplementary Data 4). Those terms accounted for 7.6% (192 genes) of the DEGs. Pearson correlation analysis revealed that they are significantly co-expressed, even with a multiple correction threshold FDR < 0.01 (Figure 4 and Supplementary Data 5). In addition to GO biological processes, we also identified several overrepresented KEGG pathways (Supplementary Data 4), including ribosome, cytokine–cytokine receptor interaction, cyclic AMP signaling pathway, and calcium signaling pathways.

**Figure 5:**
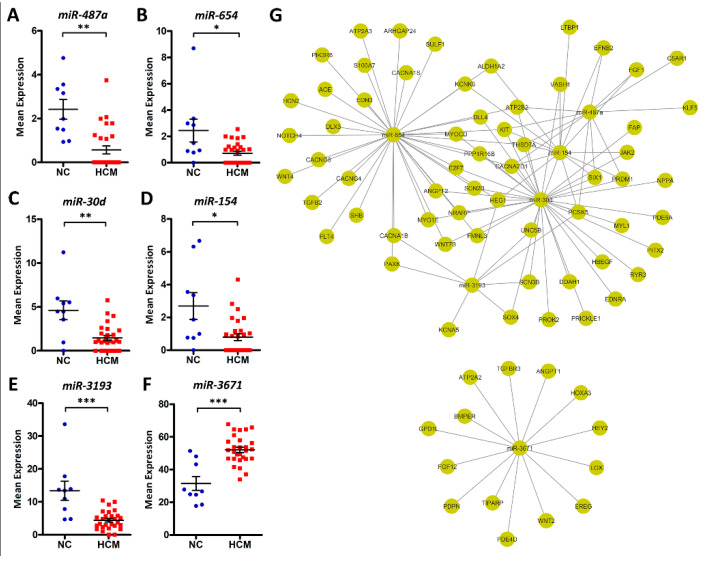
Differentially expressed microRNAs (miRNAs) between HCM patients and normal controls. (**A**–**F**) Dot plots of *miR-487a*, *miR-654*, *miR-30d*, *miR-154*, *miR-3193*, and *miR-3671* expression levels in NC and HCM patients, *FDR < 0.05, **FDR < 0.01, ***FDR < 0.001. (**G**) Predicted miRNA-gene pairs with miRWalk2, which implements four miRNA-gene prediction algorithms. Target pairs for which more than two algorithms had signals are included. Seventy-five cardiovascular-related DEGs were targeted by the above miRNAs, with these genes demonstrating inverse expression patterns compared to their corresponding miRNAs.

There are only six miRNAs that showed significant differences, with *miR-487a*, *miR-654*, *miR-30d*, *miR-154*, and *miR-3193* being downregulated and *miR-3671* being upregulated in HCM patients (Figure 5A–F). Among them, *miR-30d* and *miR-154* have been shown to play regulatory roles in myocardial fibrosis [13,14]. Next, their targeting genes were predicted using miRWalk2. A total of 720 differentially expressed protein-coding genes (Supplementary Data 6) were targeted by the above miRNAs, with these genes demonstrating inverse expression patterns compared to their corresponding miRNAs. Among them, 75 are involved in cardiovascular system regulation (Figure 5G). In addition, we also performed the Pearson correlation analysis. The results demonstrated that the percentages of significantly correlated (*p* < 0.05) candidate pairs ranged from 25.5% (*miR-154*) to 76.5% (*miR-3671*). 

In order to dissect the functional roles of the differentially expressed lncRNAs, we retrieved a total of 1482 protein-coding genes near (< 0.5 Mb) those lncRNAs (Supplementary Data 7), including cardiomyopathy-related genes: *TTN*, *ACTC1*, *TPM1*, *JPH2*, *ANKRD1*, *DTNA*, *TMPO*, *FHL2*, *CTNNA3*, *GATA4*, *LAMA2*, *PRDM16*, *POLG*, etc. Further GO enrichment analysis illustrated that these genes are significantly enriched in cardiovascular-related categories (Table 4), including 95 genes involved in cardiovascular system development (GO:0072358), 75 genes related to heart development (GO:0007507), and 31 genes critical to cardiac muscle tissue development (GO:0048738).

### 2.4. Co-Expression Network Analysis

**Figure 6:**
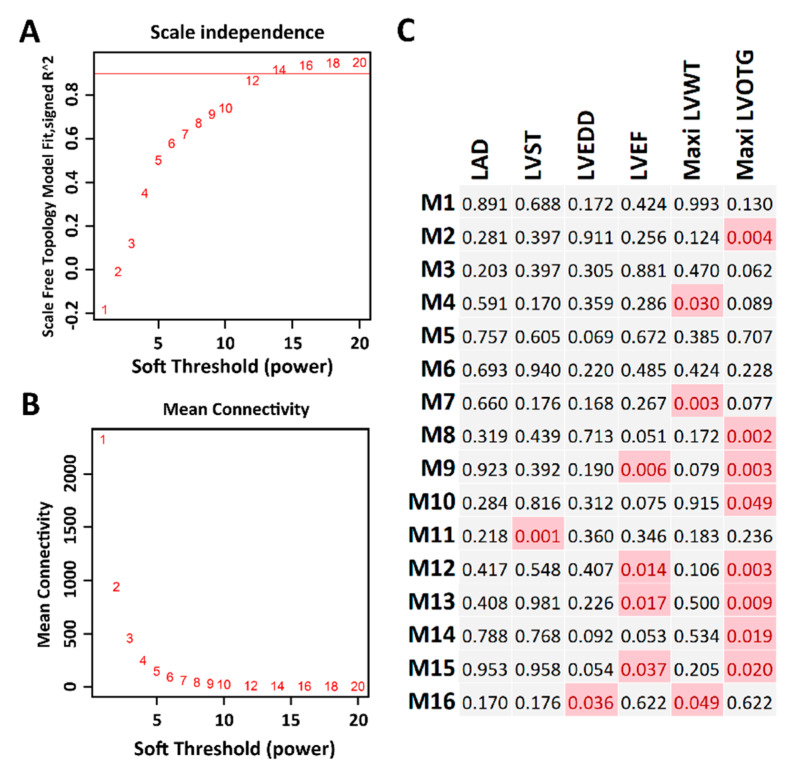
Co-expression network analysis. (**A**) The soft thresholding index R^2^ (*y*-axis) as a function of the different thresholding power β (*x*-axis). (**B**) Mean connectivity (*y*-axis) as a function of the power β (*x*-axis). (**C**) Sixteen co-expression modules identified from the myocardial RNA-seq dataset. Each cell represents the correlation coefficient *p*-value computed from correlating the module eigengenes to the clinical traits (columns). Cells filled with red represent significant associations between modules and traits (*p* < 0.05).

To gain insight into the functional organization of the transcriptomes from myocardial tissue, we conducted gene co-expression network analysis with WGCNA. By applying data transformation and filtration according to the WGCNA pipeline (see the “Materials and Methods” section), 8417 protein-coding genes were used for constructing gene co-expression networks. With the soft-thresholding power β = 12 determined by scale-free topology (Figure 6A,B), we identified a total of 16 modules (Figure 6C). The module size ranged from 50 genes in module M11 to 2207 genes in module M8. In addition, by performing correlation analysis between the module module eigengenes (MEs) and clinical traits (Table 1), several modules were identified to be associated with HCM clinical traits (Figure 6C), including module M14 and M16, which contain *MYH7* and *MYBPC3*, respectively. 

### 2.5. PPI-Subnetwork Analysis for MYH7 and MYBPC3 Modules

**Figure 7:**
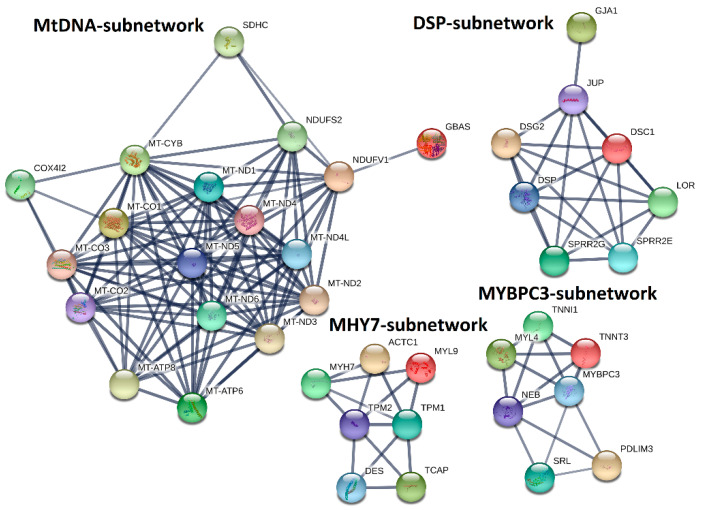
MtDNA-, DSP-, MYH7-, and MYBPC3-subnetworks constructed by integrating co-expression and protein–protein interaction (PPI) network analyses. MHY7- and MYBPC3-involved co-expression modules (M14 and M16) identified by co-expression analysis were used to construct PPI-subnetworks from the STRING database (https://string-db.org/) with the Markov Cluster (MCL) clustering method, for which the inflation parameter was set to 3.

Next, we explored the PPIs for the genes in the MHY7- and MYBPC3-involved modules (M14 and M16). By performing MCL clustering, we highlighted four subnetworks that could play significant roles in HCM, including one mtDNA-subnetwork and three cardiomyopathy-gene-centered networks: MYBPC3-, MYH7-, and DSP-subnetworks (Figure 7). Next, we performed Pearson correlation analysis for each subnetwork gene against to the HCM phenotype. This identified half of them (18 out of 39 genes), showing significant correlations (*p* < 0.05) with the measured phenotypes (Supplementary Data 8), including *NDUFS2*, *DSP*, *JUP*, *TNNI1*, etc. These genes could be directly associated with the phenotypes, while the rest may modulate the HCM phenotypes by interacting with those 18 genes.

Besides the subnetwork hub genes, other genes were also found to be associated with cardiomyopathy. For instance, *TPM1*, and *ACTC1*, which are critical for the maintenance of sarcomere thickness [15,16], have been associated with familial HCM [17,18,19]. Truncating mutations in *DES* are associated with inherited skeletal and cardiac myopathies [20]. *TCAP* is required for sarcomerogenesis in striated muscles, and mutations in *TCAP* have been identified in patients with HCM and dilated cardiomyopathy (DCM) [21]. Patients with heterozygous mutations in *JUP* have been diagnosed with HCM [22,23], and reduced cardiac *DSG2* levels appear to be specifically associated with arrhythmogenic right ventricular cardiomyopathy (ARVC) [24]. For the mtDNA-subnetwork, mutations of *MT-ND1*, *MT-ND2*, *MT-ND3*, *MT-ND4*, *MT-ND4L*, *MT-ND5*, *MT-ND6*, *MT-CO1*, *MT-CO2*, *MT-CO3*, *MT-ATP6*, *MT-ATP8*, and *MT-CYB* have been identified in cardiomyopathy patients [25,26,27,28]. Levels of *NDUFV1*, a component of complex I in the electron transport chain, are significantly decreased in the myocardium of DCM patients, and *NDUFS2* gene mutations cause complex I deficiency with associated DCM [29,30]. 

## 3. Discussion

### 3.1. Pathogenic Variant Analysis and Mechanisms of Variant Synergism 

In the current study, nine HCM patients were identified with pathogenic *MYH7* variants and nine with pathogenic *MYBPC3* variants. One patient (HCM437) had both *MYH7* and *MYBPC3* variants, along with one in *COX15*, whose function appears to be essential for cytochrome c oxidase (COX) biogenesis in the electron transport chain (ETC). Mutations in this gene are associated with Leigh syndrome and COX deficiency with infantile HCM, and the presence of this variant is interesting in the context of the enriched mitochondrial PPI-subnetwork (Figure 7) [31]. All of the *MYH7* mutations were missense variants, while the *MYBPC3* mutations were frameshift, nonsense, or splice variants. Missense, rather than truncating, variants in *MYH7* and truncating, rather than missense, variants in *MYBPC3* are well-documented to be the mutational classifications associated with HCM, instead of other cardiomyopathy subtypes [32,33].

A single variant in *NEXN*, *MYH7*, *NEBL*, and *DSP* was replicated in three, two, two, and two HCM patients, respectively. Importantly, the *NEXN* and *DSP* variants have been predominantly classified as “likely benign” within the NCBI ClinVar database; the *NEBL* mutation is described as a variant of uncertain significance. For patients HCM282 and HCM552, the *NEXN* and *NEBL* variants, respectively, occur in isolation, which may mean that the phenotype results from the presence of these variants in conjunction with unidentified modifier variants. The *NEBL* variant has been found in isolation in one Japanese patient with DCM and in conjunction with another cardiomyopathy-associated variant in a Japanese HCM patient [34]. 

Both of the patients with the *DSP* variant (HCM562 and HCM591) have another cardiomyopathy gene identified. Desmoplakin, which is encoded by *DSP*, is a desmosomal plaque protein, and variants in this gene are implicated in ARVC [35]. *PSEN2* mutations, like the one in HCM591, have been reported in association with familial Alzheimer’s disease and DCM, but not HCM, and those associated with DCM impair calcium signaling and handling [36]. The presenilin complex proteolytically processes desmoglein-2 (*DSG2*), another component of the desmosome, and protein networks implicate gene–gene interactions between the presenilin components and a number of desmosomal proteins, including desmoplakin [37]. This provides a mechanism for how these variants may operate synergistically to produce HCM, and the *DSP* variant may be a pro-HCM modifier gene. Overall, the *NEXN*, *NEBL*, and *DSP* mutations may represent important modifier variants, as they are disproportionately represented in this sample population.

The remaining patients without an identified pathogenic *MYBPC3* or *MYH7* variant had mutations in *ABCC9*, *SGCD*, *TTN*, *CSRP3*, and *PLN*. Variants in *ABCC9*, which encodes an ATP-sensitive K^+^ channel, have been implicated in familial atrial fibrillation, sporadic DCM, and hypertrichotic osteochondrodysplasia. DCM variants have been described in exon 38 and likely modify a nucleotide-binding domain in the SUR2A portion of the channel, altering overall channel gating [38]. HCM541′s variant in *ABCC9* is found in exon 24, which corresponds to the SunT domain of the protein (not a transmembrane region), and may represent an HCM mutational hotspot.

*SGCD* encodes the δ component of the sarcoglycan complex, and mutations in this gene are associated with autosomal recessive limb-girdle muscular dystrophy type VI and a mild autosomal dominant DCM phenotype [39,40]. The sarcoglycan complex interacts with the dystrophin complex, which is in turn composed of dystrophin and dystroglycan subunits. The disruption of sarcoglycan components has been linked with a reduced expression of α-dystroglycan at the sarcolemma, and overall dystroglycan knockout has been associated with disruptions in titin expression [41]. Encoded by *TTN*, titin maintains the structure of the sarcomeric unit in both skeletal and cardiac muscle during contraction and interacts with actin filaments and myosin [42]. While both of the variants identified in HCM493 are categorized as “likely benign”. The *SGCD* mutation may further disrupt titin networks that are already disorganized by the *TTN* variant itself and thus work synergistically to produce HCM.

*CSRP3* encodes an LIM domain-containing scaffolding protein that maintains the structure of the Z-disk and the costamere and is implicated in actin remodeling and cardiomyocyte differentiation. Mutations in this gene are associated with both familial HCM and DCM [43,44]. HCM395′s unique mutation has not been reported previously. This stop-loss variant extends the protein by 33 amino acids. A similar variant that converts the termination codon to an arginine residue (rather than a cysteine residue) and promotes a 33 amino acid extension has been labeled as a variant of uncertain significance. Further studies are needed to determine if these additional residues alter protein function, structure, and/or the rate of degradation.

Produced by *PLN*, phospholamban is a substrate of protein kinase A (PKA), a cAMP dependent kinase, in cardiomyocytes. The phosphorylation of phospholamban by PKA relieves its inhibition of ATP2A2, a sarcoplasmic reticulum Ca^2+^-ATPase, allowing for the sequestration of calcium ions and cardiac muscle relaxation in diastole [45,46]. Heterozygous *PLN* variants, like the unreported one found in HCM486, have been identified in patients with HCM; the *PLN* inheritance pattern for DCM is less clear [46,47]. Interestingly, this gene participates in both cAMP and calcium signalling pathways, reflecting the significant KEGG pathway enrichment mentioned above (Supplementary Data 4).

### 3.2. HCM Phenotypic Variability from Multi-Gene Modification and Differential Gene Expression

While HCM has been attributed to monogenic perturbations in sarcomeric genes, like *MYBPC3* and *MYH7*, there is often dramatic phenotypic variability, even within a single family, with some family members having other cardiomyopathy subtypes, including DCM and ARVC, or hybrid phenotypes. This variability is most likely attributable to the presence of multiple modifier variants, and hints at the concept that cardiomyopathy is a polygenic trait. 

Beyond the single gene and synergistic modifier gene perspectives, pathway perspectives illustrate the idea that even single gene mutations may in turn modify other genes in a specific pathway or network. The differences in expression levels of *ANKRD1*, *FHL2*, and *TGFB3* between the HCM patients and normal controls demonstrated this idea. Mutations in *FHL2* have been implicated in HCM, so the reduced expression of these genes in HCM hearts mirrors actual inherited modifications or deficiencies in these proteins [48,49,50]. Other studies have demonstrated that the elevation of *ANKRD1* expression in DCM heart samples correlates with the progression of heart failure, and this reflects the increased expression that we saw in the HCM patients [51]. Finally, HCM is characterized by cardiac fibrosis, and *TGFB3* is an important driver of the fibrosing process [52]. This is illustrated in the elevation of *TGFB3* expression in our HCM samples; likewise, missense *TGFB3* mutations have loosely been associated with HCM itself [53]. 

### 3.3. HCM Modulation with Noncoding RNAs

As post-transcriptional regulators, both miRNAs and lncRNAs can interfere with gene expression [54,55]. We explored the miRNA and lncRNA expression profiles of HCM patients and healthy controls. Six miRNAs (*miR-487a*, *miR-654*, *miR-30d*, *miR-154*, *miR-3193* and *miR-3671*) were observed to be significantly up- or down-regulated in HCM patients. Among them, Serum *miR-30d* has been reported to be associated with acute heart failure [56] and considered as a biomarker for diffuse myocardial fibrosis in HCM patients [13]. In addition, the expression of *miR-30d-5p* was decreased 1.58-fold in patients with ST-elevated myocardial infarction [57]. *MiR-154* has been reported to promote myocardial fibrosis through β-catenin signaling pathways [14,58]. It is worth noting that the 75 targeted genes in the miRNA-gene network (Figure 5G) are all involved in the cardiovascular system, suggesting those miRNAs may modulate cardiac function by regulating their expression levels. For example, the target gene *FGF1*, known as acidic fibroblast growth factor, shares regulation by *miR-487a* and *miR-154*. A pathophysiologic role for fibroblast growth factors in idiopathic cardiomyopathy has been implicated in the past [59]. Additionally, several voltage-gated calcium channels genes (*CACNA2D1*, *CACNA1B*, *CACNA1S*, *CACNG4*, and *CACNG8*) are also targeted by those miRNAs. It has been reported that mutations in *CACNA2D1* cause a variant of short QT syndrome, which is associated with sudden cardiac death in humans [60]. 

Several studies have demonstrated the importance of lncRNAs in the cardiovascular system [61,62]. We retrieved a total of 1482 protein-coding genes near the differentially expressed lncRNAs. A few lncRNAs are located in the introns of cardiomyopathy-related genes (e.g., *TTN*, *ACTC1*, *TPM1*, *JPH2*, *ANKRD1*, *DTNA*, *TMPO*, and *FHL2*) or physically close to these genes. The lncRNA-annotated protein-coding genes are enriched in the GO terms of heart development and cardiomyocyte differentiation (Table 4), while the enriched terms for the differentially expressed protein-coding genes centered more so on cardiac physiology (contractility, heart rate, and conduction; Figure 3E). This illustrates the idea that HCM can represent both an inherited defect in myocardial development (that may or may not be immediately clinically apparent) and a pathologic process of ventricular remodeling that occurs over an extended period of time due to impaired cardiac, or even renal, function. 

### 3.4. HCM Modulation with Gene Networks

Differential expression analysis usually focuses on individual gene perturbations, which cannot dissect their potential interactions. Furthermore, genes with relatively modest expression changes may also contribute to the phenotypes of interest. However, this information was usually underestimated or missed from differential expression analysis. Network-based analysis provides an alternative way to leverage this gap by grouping a subset of tightly co-expressed genes together, which can be defined as a module or network. In this study, we performed gene co-expression network analysis with WGCNA and PPI analysis with STRING. Our results highlighted four subnetworks, mtDNA-, MYBPC3-, MYH7-, and DSP-subnetworks, that may be critical to the progression of HCM. Besides the hub genes of *MYBPC3*, *MYH7*, and *DSP*, these subnetworks also include several genes that have been implicated in cardiomyopathy, such as *TPM1*, *ACTC1* [17,18,19], *DES* [20], *TCAP* [21], *JUP* [22,23], and *DSG2* [24].

In addition, our gene network analysis also highlighted the importance of mitochondrial genes (mtDNA-subnetwork) in HCM. However, those genes are largely neglected on HCM genetic screening panels. Mutations in sarcomeric genes are associated with inefficient energy utilization [63]. When these variants are present in conjunction with heterozygous oxidative phosphorylation mutations, compensations in energy production may not be possible and may contribute to the HCM phenotypes. This will require further elucidation, but screening for these genes may aid in the identification of a genetic cause of HCM in patients with mutations not included on typical HCM panels and can supplement the landscape of genetic modifiers in HCM.

### 3.5. Novelty and Limitations of This Study

As the original research of this RNA-seq data [12] was reported as data descriptor, with the major findings of this dataset is to be of high quality to perform downstream analysis such as the identification of DEGs. Compared with the original findings, our research highlighted several novel findings. First, we performed a variant calling from this RNA-seq data, our results not only confirmed the *MYH7* and *MYBPC3* pathogenic variants that have been identified in the original research, but also discovered eight candidate variants in six genetically undiagnosed patients. In addition, we also found most of the patients carry muti-genic variants, even for the *MYH7* and *MYBPC3* diagnosed patients, suggesting that a heterogeneous phenotype could exist among those patients. Second, we identified thousands of differentially expressed coding and noncoding genes. For the coding genes, we performed functional enrichment analysis, which highlighted that those genes significantly involved in regulating cardiovascular system development and cardiac physiology (192 genes, Figure 4). We found six differentially expressed miRNAs (Figure 5A–F) that could regulate HCM progression by targeting 75 cardiovascular-function-related genes (Figure 5G). Additionally, by co-locating the differentially expressed lncRNAs and protein-coding genes, we highlighted that several lncRNAs could relate to cardiac function, since they are co-located with the well-known cardiomyopathy-related genes (Table 3). Therefore, our analysis not only identified the DEGs, but also reduced the candidates from thousands of DEGs to a few dozen by integrating no-coding gene annotation and gene function enrichment analysis. Last, we performed gene network analysis based on WGCNA and PPI. This resulted in four subnetworks that are strongly correlated to the HCM clinical traits (Figure 6 and Figure 7). Those subnetworks not only include known genes implicated in HCM, but also highlighted several genes with an unknown function in HCM that need to be further deciphered (Figure 7).

There are several limitations for our studies. First, calling variants from RNA-seq is confronted with several issues, due to the complexity of the transcriptome. In order to avoid false positives/negatives, we applied SNP and Indel calling with the recommendation of the Broad Institute’s GATK best practices workflow. In addition, for the candidate variants, we also “eye” inspected the alignment to avoid false positive/negatives raised by too much complexity around the variant region. Further, our results confirmed the *MYH7* and *MYBPC3* pathogenic variants that have been identified in the original paper, highlighting the variant-calling pipeline and that the results are reliable. Second, the clinical measurement of the studied sample is not fully characterized (such as morphological or magnetic resonance imaging data). This limited our genotype–phenotype correlations, especially for evaluating the clinical outcome differences between the patients carry muti-genic and single gene mutation. Third, the larger sample size would be beneficial for covering broader variants’ spectrum and increase the statistical power for differential expression analysis and gene co-expression network construction. 

## 4. Materials and Methods 

### 4.1. RNA-Seq Data

The RNA-seq data of SRP186138 [12] were downloaded from the NCBI Sequence Read Archive database (https://www.ncbi.nlm.nih.gov/sra/SRP186138). This data set contains 28 myocardial samples from HCM patients and 9 samples from healthy donors. The detailed clinical characteristics of the studied samples are listed in Table 1.

### 4.2. Read Mapping and Variant Calling

The raw reads for each sample first underwent adaptor trimming and filtering with the fastp software [64], allowing for reads containing over 80% bases with a quality greater than 20 to be included. Read mapping and variant calling were done according to the Broad Institute’s GATK [65] best practices workflow for SNP and Indel calling on RNAseq data (https://software.broadinstitute.org/gatk/documentation/article.php?id=3891). Briefly, paired-end reads (PE300) were mapped onto the human reference genome (GRCh38) using the STAR 2-pass alignment method [66]. The first-pass alignment was used to generate the junction file. Then, the second-pass alignment was conducted with the junctions found in the first-pass mapping to produce the final alignments. After that, Picardtools (http://broadinstitute.github.io/picard/) was used to add read groups, sort, mark duplicates, and create index. Then, the SplitNCigarReads function, implemented in GATK, was used to split reads into exon segments. Finally, variant calling and filtering were done with HaplotypeCaller and VariantFiltration, respectively.

### 4.3. Functional Variants and Gene Prioritization 

The variant call was first annotated with wANNOWAR [67]. Then, the following strategies were applied to identify potential functional variants and genes: 1) variants only present in the 28 HCM patients, but not in the 9 controls; 2) variants classified as nonsynonymous, stop-gain, stop-loss, frameshift, or splice altering; 3) variants with a minor allele frequency less than 0.01 in 1000G, ExAC, ESP6500, or gnomeAD data sets; 4) variants predicted to be deleterious by no less than five of the following algorithms—SIFT, Polyphen2, LRT, MutationTaster, FATHMM, PROVEAN, MetaSVM, and CADD, and 5) variants only in the 73-gene HCM panel listed in Supplementary Data 1.

### 4.4. Differential Expression Analysis

A comparison of HCM patients with normal controls was used to identify differentially expressed genes (DEGs). Gene-level read counts were obtained using featureCount version 0.6.1 with Ensembl annotation of the GRCh38 reference genome, which includes both coding and non-coding genes. Raw counts were analyzed with DESeq2 version 1.10.1 [68] to identify DEGs. *p*-values were adjusted with the false discovery rate (FDR) based on Benjamini and Hochberg’s method [69], and DEGs were defined as FDR < 0.05 and fold change > 1.5. 

### 4.5. MiRNA Target Prediction

MiRNAs are a class of non-coding small RNAs (~22 nucleotides), which are widely found in plant and animal cells. Cleavage of mRNA transcripts or the inhibition of translation initiation is achieved by inaccurate complementary base pairing with the target gene [70]. To date, several algorithms have been proposed and applied to predict miRNAs targets [71]. In this study, we used miRWalk 2 [72], which implements the four miRNA-gene prediction programs “miRWalk,” “miRanda”, “RNA22”, and “Targetscan” to predict miRNA gene targets. We considered the positive target pairs for which more than two algorithms had signals.

### 4.6. LncRNA Annotation

LncRNAs are a class of non-coding RNAs that are greater than 200 nucleotides in length [73]. Although most of them have not been functionally characterized, studies have shown that they play significant roles in gene expression regulation, especially for neighboring genes, at various levels, such as transcriptional and post-transcriptional regulation [73,74]. In order to interpret the potential functional roles of the differentially expressed lncRNAs, their neighbor genes (< 0.5 Mb) were extracted using bedtools [75] and further explored by performing gene set enrichment analysis. 

### 4.7. Co-Expression Network Analysis

Gene co-expression networks was constructed using the WGCNA package in R [76], according to the tutorials written by Peter Langfelder and Steve Horvath (https://horvath.genetics.ucla.edu/html/CoexpressionNetwork/Rpackages/WGCNA/Tutorials/). We first normalized the raw read count with the Transcripts Per Million (TPM) method and then removed genes whose expression was consistently low (TPM < 10 in more than 90% of the samples). The filtered data was log-transformed with log2(x+1), which was used as input for WGCNA analysis. Based on the scale-free topology, threshold power β = 5 was used for constructing the adjacency matrix and the Topological Overlap Matrix. Then, genes were aggregated into modules with hierarchical clustering and further refined using the dynamic tree cut algorithm. In order to identify the clinical-trait-associated modules, we performed Pearson’s correlation coefficient analysis between the traits and MEs, which are defined as the first principal component of a given module. 

### 4.8. PPI Network Analysis

The PPI network analysis of module genes was based on the protein interaction information retrieved from the online database STRING (https://string-db.org/) [77]. To define subnetworks, we first only selected genes with a high confidence interaction score (> 0.7). Those highly confident interactions were further clustered into subnetworks with the MCL clustering method, for which the inflation parameter was set to 3.

### 4.9. Gene Over-Representation Analysis

Gene overrepresentation analysis for GO (biological process) and KEGG pathway was analyzed with WebGestalt (http://www.webgestalt.org/) with default parameters [78]. The human genome was used as a reference gene set, and the minimum number of genes for a category was set to 5 for both analyses. The Benjamini and Hochberg correction [69] was used for multiple test correcting. A threshold of FDR < 0.05 was used to determine the significantly enriched terms.

### 4.10. Data Statement

Raw RNA-seq data had been deposited in the NCBI Sequence Read Archive database (SRA; https://www.ncbi.nlm.nih.gov/sra/) under the accession number SRP186138 [12].

## 5. Conclusions

In summary, by analyzing myocardial RNA-seq data from HCM and control patients, we provide evidence that HCM is a complex genetic disorder, with pathogenic and modifier protein-coding mutations, differential gene expression, and non-coding RNA modulation contributing to extensive phenotypic variability. In addition, this work highlights the importance of integrative analysis with gene co-expression and protein–protein interaction networks for the further identification of functional HCM genes within gene subnetworks. Further elucidation of HCM genes, whether at the level of the single protein-coding gene or the gene network, will lead to improvements in genetic screening for HCM patients without an identified genetic cause and to the development of new, personalized therapeutics.

## Figures and Tables

**Table 1:** Clinical characteristics of the studied samples [12].

Sample ID	Group^#^	Sex	Age	Smoking	LAD (mm)^a^	LVST (mm)^b^	LVEDD (mm)^c^	LVEF (%)^d^	Maxi LVWT (mm)^e^	Maxi LVOTG (mmHg)^f^
HCM420	GENETUN	male	32	NA	41	14	44	76	18	55
HCM405	GENETUN	female	31	NO	42	14	47	78	16	126
HCM541	GENETUN	male	38	NA	47	22	46	71	25	96
HCM493	GENETUN	male	40	NA	40	15	45	75	17	95
HCM273	GENETUN	male	30	NO	38	15	33	50	21	30
HCM269	GENETUN	male	25	NO	53	29	51	63	38	56
HCM395	GENETUN	male	20	NO	49	19	43	60	22	78
HCM282	GENETUN	male	48	YES	49	21	49	65.3	23	75
HCM591	GENETUN	male	42	NA	39	21	45	70	32	53
HCM552	GENETUN	male	32	NA	NA	NA	NA	75	21	100
HCM515	*MYBPC3*	male	21	YES	39	18	50	75	25	84
HCM504	*MYBPC3*	male	25	YES	39	25	39	75	28	66
HCM498	*MYBPC3*	male	39	YES	38	20	42	72	21	55
HCM486	*MYBPC3*	female	36	NA	45	16	35	75	20	100
HCM460	*MYBPC3*	male	43	YES	45	16	45	65	19	61
HCM439	*MYBPC3*	female	47	NA	36	15	38	80	18	103
HCM437	*MYBPC3*	male	30	YES	54	19	47	78	26	54
HCM429	*MYBPC3*	female	36	NA	48	17	42	78	18	70
HCM533	*MYBPC3*	male	47	YES	62	24	50	75	29	118
HCM518	*MYBPC3*	female	31	NA	37	22	38	68	26	105
HCM506	*MYH7*	male	19	YES	48	20	43	75	21	59
HCM562	*MYH7*	female	36	YES	36	0	36	66	21	70
HCM490	*MYH7*	female	41	NA	44	15	39	58	21	63
HCM491	*MYH7*	female	30	NA	44	23	32	75	28	64
HCM456	*MYH7*	male	28	NA	51	15	45	69	20	90
HCM483	*MYH7*	male	37	YES	57	23	40	72	26	70
HCM431	*MYH7*	male	24	NA	57	18	49	80	21	80
HCM443	*MYH7*	female	28	NA	46	17	44	69	18	68
sc5-LV	NORMAL	female	NA	NA	NA	NA	NA	NA	NA	NA
sc2-LV	NORMAL	male	NA	NA	NA	NA	NA	NA	NA	NA
sc6-LV	NORMAL	male	NA	NA	NA	NA	NA	NA	NA	NA
N105-LV	NORMAL	male	NA	NA	NA	NA	NA	NA	NA	NA
N104-LV	NORMAL	male	NA	NA	NA	NA	NA	NA	NA	NA
ND2	NORMAL	male	NA	NA	NA	NA	NA	NA	NA	NA
ND1-LV	NORMAL	male	NA	NA	NA	NA	NA	NA	NA	NA
N102-LV	NORMAL	male	NA	NA	NA	NA	NA	NA	NA	NA
N103-LV	NORMAL	male	NA	NA	NA	NA	NA	NA	NA	NA

^#^: GENETUN, genetically undiagnosed HCM patient; *MYBPC3*, HCM patient with mutation in *MYBPC3*; *MYH7*, HCM patient with mutation in *MYH7*; NORMAL, normal heart; ^a^: left atrial diameter; ^b^: left ventricular septal thickness; ^c^: left ventricular end-diastolic; ^d^: left ventricular ejection fraction; ^e^: maximum left ventricular wall thickness; ^f^: maximum left ventricular outflow track gradient at rest or after exercise; NA: not available.

**Table 2:** Summary statistics of RNA-seq data.

Sample ID	Raw Reads	HQ Reads	HQ Reads (%)	Mapping Rate (%) ^#^	Total Variant	Exonic Variant
HCM420	117,254,002	110,409,284	94.16	96.87	742,007	34,208
HCM405	96,660,588	90,958,152	94.10	96.72	697,916	32,501
HCM541	118,364,902	112,199,122	94.79	96.56	748,918	34,438
HCM493	135,595,138	127,235,398	93.83	96.67	802,057	35,285
HCM273	100,383,614	94,562,944	94.20	96.10	674,997	32,320
HCM269	104,573,612	98,483,828	94.18	96.69	657,403	30,937
HCM395	101,463,898	94,778,784	93.41	96.50	718,773	32,792
HCM282	135,385,378	128,496,976	94.91	96.41	835,560	36,065
HCM591	117,087,250	110,633,048	94.49	96.33	742,357	33,694
HCM552	110,633,048	97,644,508	88.26	96.57	714,914	32,157
HCM515	126,385,206	120,132,036	95.05	96.53	797,070	35,707
HCM504	126,614,812	120,041,188	94.81	96.46	747,293	34,481
HCM498	118,114,640	110,661,262	93.69	96.18	745,414	33,633
HCM486	102,005,260	95,821,344	93.94	96.76	688,159	32,517
HCM460	83,321,826	78,346,916	94.03	97.01	641,381	30,658
HCM439	115,966,430	108,531,416	93.59	96.48	777,052	34,950
HCM437	114,409,150	107,630,394	94.07	96.59	718,353	33,216
HCM429	107,327,856	100,460,638	93.60	96.51	781,013	34,514
HCM533	113,801,296	107,068,190	94.08	96.47	765,001	34,584
HCM518	123,140,430	116,174,372	94.34	96.54	821,103	36,229
HCM506	104,464,396	98,442,104	94.24	96.88	721,500	33,453
HCM562	119,176,332	113,518,840	95.25	96.63	782,848	34,382
HCM490	109,178,274	102,325,212	93.72	96.79	728,218	33,873
HCM491	123,213,328	115,698,990	93.90	95.97	756,623	34,040
HCM456	124,188,740	116,826,920	94.07	96.65	793,088	35,368
HCM483	132,375,212	125,845,538	95.07	96.52	838,862	35,806
HCM431	95,467,544	91,611,504	95.96	96.60	701,266	33,263
HCM443	103,385,170	96,969,874	93.79	96.18	707,524	33,016
sc5-LV	115,206,460	109,427,776	94.98	94.80	731,586	35,144
sc2-LV	119,628,518	113,668,538	95.02	96.52	747,079	34,629
sc6-LV	133,156,688	126,807,678	95.23	95.59	789,274	37,289
N105-LV	125,064,692	118,533,240	94.78	96.39	830,057	35,975
N104-LV	151,765,118	143,413,882	94.50	96.50	930,969	37,875
ND2	115,981,584	110,192,388	95.01	94.90	649,196	33,173
ND1-LV	111,029,746	105,848,092	95.33	95.66	715,284	33,920
N102-LV	111,141,904	102,446,288	92.18	96.59	754,272	33,343
N103-LV	146,673,012	138,682,818	94.55	96.50	864,176	35,856

**^#^**: the percentage of uniquely mapped reads to the total high-quality (HQ) reads.

**Table 3:** Lists of candidate variants prioritized from HCM patients.

Group	Sample ID	Gene	dbSNP	Variant Type	AAChange^#^
GENETUN	HCM541	*ABCC9*	rs763968252	nonsynonymous	NM_005691:exon24:c.T2935C:p.W979R
	HCM493	*SGCD*	rs397516338	nonsynonymous	NM_001128209:exon8:c.A845G:p.Q282R
		*TTN*	rs146400809	nonsynonymous	NM_133378:exon126:c.C28730T:p.P9577L
	HCM395	*CSRP3*	NA	stop loss	NM_003476:exon7:c.A585C:p.X195C
	HCM282	*NEXN*	rs146245480	nonsynonymous	NM_001172309:exon7:c.C643T:p.R215C
	HCM591	*DSP*	rs116888866	nonsynonymous	NM_001008844:exon24:c.G6119A:p.R2040Q
		*PSEN2*	rs574125890	nonsynonymous	NM_000447:exon8:c.G640T:p.V214L
	HCM552	*NEBL*	rs75301590	nonsynonymous	NM_006393:exon6:c.G561C:p.Q187H
MYBPC3	HCM515	*MYBPC3*	rs869025465	frameshift deletion	NM_000256:exon13:c.1153_1168del:p.V385Mfs*15
		*NEXN*	rs146245480	nonsynonymous	NM_001172309:exon7:c.C643T:p.R215C
		*LDB3*	rs566463138	nonsynonymous	NM_001080114:exon10:c.T1367G:p.M456R
		*TNNT2*	rs141121678	nonsynonymous	NM_001001432:exon14:c.G839A:p.R280H
	HCM504	*MYBPC3*	NA	frameshift deletion	NM_000256:exon28:c.3018delC:p.W1007Gfs*12
		*TTN*	rs118161093	nonsynonymous	NM_003319:exon27:c.G5602A:p.A1868T
		*TTN*	rs139517732	nonsynonymous	NM_001256850:exon3:c.G160A:p.V54M
	HCM498	*MYBPC3*	NA	stop gain	NM_000256:exon5:c.G587A:p.W196X
		*ACTN2*	rs376144003	nonsynonymous	NM_001103:exon11:c.T1162A:p.W388R
		*PSEN2*	NA	nonsynonymous	NM_000447:exon10:c.C902A:p.T301K
	HCM486	*PLN*	NA	nonsynonymous	NM_002667:exon2:c.T106C:p.C36R
	HCM460	*MYBPC3*	rs730880576	stop gain	NM_000256:exon26:c.G2748A:p.W916X
		*LDB3*	rs397517221	nonsynonymous	NM_001080114:exon2:c.C236T:p.T79I
		*TTN*	rs199932621	nonsynonymous	NM_003319:exon186:c.G75632A:p.R25211Q
	HCM439	*MYBPC3*	rs397516073	splicing	NA
		*LAMA4*	rs3752579	nonsynonymous	NM_001105206:exon12:c.T1475A:p.L492H
		*PSEN2*	NA	nonsynonymous	NM_000447:exon13:c.G1234A:p.A412T
	HCM437	*MYBPC3*	NA	frameshift insertion	NM_000256:exon13:c.1201dupC:p.Q401Pfs*12
		*MYH7*	rs727503278	nonsynonymous	NM_000257:exon5:c.C427T:p.R143W
		*COX15*	rs769275933	nonsynonymous	NM_001320976:exon9:c.C584T:p.T195M
	HCM429	*MYBPC3*	NA	frameshift deletion	NM_000256:exon22:c.2237delA:p.E746Gfs*6
		*NEXN*	rs146245480	nonsynonymous	NM_001172309:exon7:c.C643T:p.R215C
	HCM533	*MYBPC3*	NA	stop gain	NM_000256:exon24:c.C2526G:p.Y842X
		*TTN*	rs549841864	nonsynonymous	NM_003319:exon167:c.C66059T:p.P22020L
	HCM518	*MYBPC3*	NA	frameshift deletion	NM_000256:exon4:c.480delG:p.P161Hfs*5
MYH7	HCM506	*MYH7*	rs121913627	nonsynonymous	NM_000257:exon16:c.G1816A:p.V606M
		*RBM20*	rs372923744	nonsynonymous	NM_001134363:exon9:c.G2201A:p.R734Q
	HCM562	*MYH7*	rs727503278	nonsynonymous	NM_000257:exon5:c.C427T:p.R143W
		*DSP*	rs116888866	nonsynonymous	NM_001008844:exon24:c.G6119A:p.R2040Q
	HCM490	*MYH7*	rs397516201	nonsynonymous	NM_000257:exon30:c.C4130T:p.T1377M
		*TTN*	rs368057764	nonsynonymous	NM_003319:exon79:c.C19652T:p.T6551M
		*TTN*	*NA*	nonsynonymous	NM_003319:exon154:c.G56641A:p.D18881N
		*TTN*	rs567446185	nonsynonymous	NM_003319:exon154:c.G46322A:p.G15441D
	HCM491	*MYH7*	rs397516127	nonsynonymous	NM_000257:exon18:c.C1987T:p.R663C
		*MYH6*	NA	splicing	NA
	HCM456	*MYH7*	rs3218714	nonsynonymous	NM_000257:exon13:c.C1207T:p.R403W
	HCM483	*MYH7*	rs121913627	nonsynonymous	NM_000257:exon16:c.G1816A:p.V606M
	HCM431	*MYH7*	rs727503246	nonsynonymous	NM_000257:exon30:c.G4066A:p.E1356K
		*PSEN1*	NA	splicing	NA
	HCM443	*MYH7*	rs121913632	nonsynonymous	NM_000257:exon20:c.G2221T:p.G741W
		*NEBL*	rs75301590	nonsynonymous	NM_006393:exon6:c.G561C:p.Q187H

#: Gene may have multiple transcript annotations, but only the first is shown here; the full list of annotations can be found in Supplementary Data 2; NA: not available.

**Table 4:** GO enrichment of long noncoding RNA (lncRNA)-annotated protein-coding genes.

GO Term	Description	Gene Count	*p*-value	FDR
GO:0072358	cardiovascular system development	95	6.21 × 10^−14^	8.38 × 10^−11^
GO:0007507	heart development	75	2.71 × 10^−10^	1.10 × 10^−7^
GO:0003007	heart morphogenesis	42	7.29 × 10^−9^	1.89 × 10^−6^
GO:0003231	cardiac ventricle development	26	1.18 × 10^−7^	1.61 × 10^−5^
GO:0003206	cardiac chamber morphogenesis	25	6.39 × 10^−7^	6.43 × 10^−5^
GO:0003205	cardiac chamber development	29	1.09 × 10^−6^	9.88 × 10^−5^
GO:0048738	cardiac muscle tissue development	31	4.73 × 10^−6^	3.00 × 10^−4^
GO:0060411	cardiac septum morphogenesis	16	5.09 × 10^−6^	3.11 × 10^−4^
GO:0003208	cardiac ventricle morphogenesis	17	7.56 × 10^−6^	4.43 × 10^−4^
GO:0035051	cardiocyte differentiation	22	2.22 × 10^−5^	1.12 × 10^−3^
GO:0003279	cardiac septum development	19	3.35 × 10^−5^	1.59 × 10^−3^
GO:0003215	cardiac right ventricle morphogenesis	8	3.44 × 10^−5^	1.62 × 10^−3^
GO:0003197	endocardial cushion development	11	8.84 × 10^−5^	3.62 × 10^−3^
GO:1905207	regulation of cardiocyte differentiation	10	2.19 × 10^−4^	7.33 × 10^−3^
GO:0003203	endocardial cushion morphogenesis	9	2.62 × 10^−4^	8.54 × 10^−3^
GO:0055007	cardiac muscle cell differentiation	17	3.08 × 10^−4^	9.57 × 10^−3^
GO:0055008	cardiac muscle tissue morphogenesis	13	3.15 × 10^−4^	9.72 × 10^−3^
GO:0060047	heart contraction	33	5.04 × 10^−4^	1.46 × 10^−2^
GO:0003015	heart process	33	6.21 × 10^−4^	1.72 × 10^−2^
GO:0035050	embryonic heart tube development	14	6.58 × 10 ^−4^	1.80 × 10^−2^
GO:0055017	cardiac muscle tissue growth	13	6.92 × 10^−4^	1.88 × 10^−2^
GO:0061323	cell proliferation involved in heart morphogenesis	6	7.55 × 10^−4^	1.98 × 10^−2^
GO:0055012	ventricular cardiac muscle cell differentiation	6	7.55 × 10^−4^	1.98 × 10^−2^
GO:1905209	positive regulation of cardiocyte differentiation	7	1.00 × 10^−3^	2.44 × 10^−2^
GO:0003170	heart valve development	9	1.27 × 10^−3^	2.97 × 10^−2^
GO:0003272	endocardial cushion formation	7	1.31 × 10^−3^	3.02 × 10^−2^
GO:0010002	cardioblast differentiation	6	1.46 × 10^−3^	3.32 × 10^−2^
GO:0060419	heart growth	13	1.59 × 10^−3^	3.52 × 10^−2^
GO:0003143	embryonic heart tube morphogenesis	12	1.73 × 10^−3^	3.78 × 10^−2^
GO:2000725	regulation of cardiac muscle cell differentiation	7	2.14 × 10^−3^	4.40 × 10^−2^
GO:0001947	heart looping	11	2.44 × 10^−3^	4.85 × 10^−2^

## Supplementary Materials

Supplementary Materials can be found at https://www.mdpi.com/1422-0067/21/9/3040/s1.

## Abbreviations

ARVC	arrhythmogenic right ventricular cardiomyopathy
DCM	dilated cardiomyopathy
DEG	differentially expressed gene
FDR	false discovery rate
GO	Gene Ontology
HCM	hypertrophic cardiomyopathy
Indel	insertion and deletion
KEGG	Kyoto Encyclopedia of Genes and Genomes
LncRNA	long noncoding RNA
MEs	module eigengenes
MiRNA	microRNA
NC	Normal control
PPI	protein-protein interaction
SNP	single nucleotide polymorphism
TPM	transcripts per million
WGCNA	weighted gene co-expression network analysis.
